# Effect of Plant Water Deficit Irrigation on the Postharvest Nutritional Quality Parameters and Antioxidant Pathway of ‘Soreli’ Kiwifruits

**DOI:** 10.3390/foods15030520

**Published:** 2026-02-02

**Authors:** Micaela Lembo, Elvira Ferrara, Danilo Cice, Roberto Forniti, Vanessa Eramo, Milena Petriccione, Rinaldo Botondi

**Affiliations:** 1 Department for Innovation in Biological, Agro-Food and Forest Systems, University of Tuscia, 01100 Viterbo, Italy; micaela.lembo@unitus.it (M.L.); forniti@unitus.it (R.F.); vanessa.eramo@unitus.it (V.E.); 2 Council for Agricultural Research and Economics (CREA)—Research Center for Olive, Fruits and Citrus Crops, 81100 Caserta, Italy; elvira.ferrara@crea.gov.it (E.F.); danilo93mhr@gmail.com (D.C.); milena.petriccione@crea.gov.it (M.P.)

**Keywords:** Soreli kiwifruit, antioxidant activity, water reduction, postharvest quality, water management, agri-food supply chain

## Abstract

This study investigated the effects of regulated deficit irrigation on quality and postharvest characteristics of ‘Soreli’ kiwifruit (*Actinidia chinensis* Planch.). Plants were irrigated at 100% (control), 80%, and 60% of the standard water supply. Fruit quality was monitored by assessing weight loss (WL), firmness, soluble solids content (SSC), and color stability. Bioactive compounds, such as polyphenols (POL), flavonoids (FLAV), ascorbic acid (AA), β-carotene (Car), and chlorophyll (Chl) content and antioxidant enzyme activities, including ascorbate peroxidase (APX), superoxide dismutase (SOD), and catalase (CAT), and the 2,2-azinobis-(3-ethylbenzothiazoline-6-sulphonic acid) (ABTS) assay were also evaluated. Results indicated that reduced irrigation at 60% of water supply enhanced antioxidant enzyme levels, without negatively affecting fruit quality parameters: greater resistance to firmness loss, higher soluble solids accumulation, and better color stability. In the early stages of cold storage, fruits under the 60% irrigation treatment showed higher POL, FLAV, and ABTS values, with polyphenols exceeding 200 mg GAE 100 g^−1^ FW and FLAV content ranging from 4.69 to 5.53 mg CE 100 g^−1^ FW. The 80% irrigation treatment showed a moderate biochemical response without altering quality. Controlled water deficit can enhance antioxidant activity and bioactive compounds, improving fruit quality and the environmental and commercial value of ‘Soreli’ kiwifruit.

## 1. Introduction

Yellow-fleshed kiwifruit (*Actinidia chinensis* Planch.) is native to China and is now grown in several countries of world including Italy, the first European kiwifruit producer with a production estimated at 523,000 tons, representing about 12% of global production [1].

Compared to green-fleshed kiwifruit, the yellow-fleshed cultivars (CV) are popular among consumers due to their sweeter taste, smooth skin, and rich nutritional profile [2], including a higher vitamin C content, excellent equilibrium between sugar and acidity, and a remarkable antioxidant pattern [3]. Recent studies have demonstrated the potential health benefits of yellow-fleshed kiwifruit, including its role in enhancing immune function, supporting digestive health, and its possible anti-inflammatory properties [4].

Among the yellow CVs, Soreli, produced in 2008 by the University of Udine (Italy), is characterized by agronomic features such as high productivity, early ripening, and large fruit size, with excellent taste and nutritional quality traits [5].

Fruit ripening is a process that involves the regulation of various biochemical and physiological traits, as well as changes in oxidative metabolism. During storage, fruits produce reactive oxygen species (ROS) such as (OH·), (·HO_2_), (O_2_^−^), and (O_3_^−^) ions, which can act as signals and secondary messengers for activating stress and defense pathways, but their accumulation can result in oxidative damage to biomolecules. The intercellular levels of ROS depend on the balance between their generation and the ability to remove them due to antioxidant pathways that include both enzymatic and non-enzymatic systems [6]. Non-enzymatic systems including ascorbic acid, polyphenols, carotenoids, anthocyanins, and other molecules work alongside enzymatic antioxidant systems characterized by key enzymes such as superoxide dismutase (SOD), peroxidases (in the ascorbate-APX, glutathione-GPX forms), and catalase [7,8,9].

It was observed that yellow-fleshed kiwifruit CVs are generally more perishable than green-fleshed ones and this can be explained by a lower activity of antioxidant enzymes, such as catalase (CAT), guaiacol peroxidase (GSH), superoxide dismutase (SOD), and ascorbate peroxidase (APX) in the yellow fruits [10]. However, the reduction in antioxidant content, the rapid softening and, in general, the rapid ripening time from harvest of ‘Soreli’ kiwifruits, makes it challenging to maintain quality and meet consumer expectations above all under inadequate postharvest storage or distribution conditions [11]. Addressing these concerns, applying several pre- or postharvest strategies is essential for enhancing the competitiveness of this kiwifruit cultivar for the market [5,12]. Some studies evaluated regulated deficit irrigation to assess the effect on plant health and fruit quality during storage [2,13,14] and to obtain sustainable horticultural management, decreasing water waste, costs, and environmental concerns [15]. Limited water use efficiency is driving growers to implement advanced irrigation and cultivation strategies that optimize water utilization and improve crop yield [16]. Regulating irrigation can affect the quality of growth and the nutritional content of fruits and vegetables [17], increasing the content of soluble solids, titratable acids, and vitamin C while improving the firmness, shape, and color of the fruit [18]. For example, Wang et al. [19] evaluated pear CVs regarding how several low drip irrigation volumes had beneficial effects on some quality parameters, such as producing a good balance between soluble solid content (SSC) and titratable acidity (TA). Other studies confirm that water deficit irrigation in kiwifruit plants can be useful to improve some fruit quality attributes [20,21]. The reduction in irrigation water in horticulture can be provided with different approaches, both as the “classic deficit irrigation strategy” that implies that water is supplied at low levels throughout the season, and as “regulated deficit irrigation”, which involves an intermittent water deficit which can be beneficial for low water use efficiency, increase water savings, and improve harvest fruit quality [15,22]. Another approach is “partial root drying” which exposes roots to alternating water cycles, reducing water loss and vegetative growth while enhancing water use efficiency and influencing both fruit yield and quality. Grapevines and several fruit plants, such as peach plants, appear to adapt well to deficit irrigation, whereas other crops, such as vegetables, often struggle due to reductions in yield and quality [22]. As a result, different irrigation management strategies can lead to notable variations in crop yield, fruit quality, and the overall reduction in water usage. In this context, irrigation with low to moderate water reduction has been shown not to induce adverse effects on vegetative or fruit growth, but rather to provide favorable conditions for supporting overall vegetative development in the ‘Soreli’ plant.

Several studies [23,24] have reported that the application of low levels of regulated deficit irrigation in yellow-fleshed kiwifruit plants positively affects several quality parameters at harvest and during storage, including higher SSC and firmer fruit texture. In kiwifruit, severe water stress under high evaporative demand may permanently impair xylem hydraulic conductivity; however, low to moderate water stress (up to −0.8 MPa) has been shown not to cause damage to the vascular system of ‘Soreli’ plants. In this cultivar, the physiological responses of the plants are not altered by the application of low to moderate regulated deficit irrigation levels, nor is fruit yield at harvest adversely affected.

This study aims to investigate the impact of different volume contents delivered for irrigation in the plant’s orchard on the postharvest fruit quality traits. To do this, the contents of bioactive compounds and the antioxidant enzymes activity of ‘Soreli’ kiwifruit during cold storage were evaluated.

## 2. Materials and Methods

### 2.1. Experimental Design

**Figure 1:**
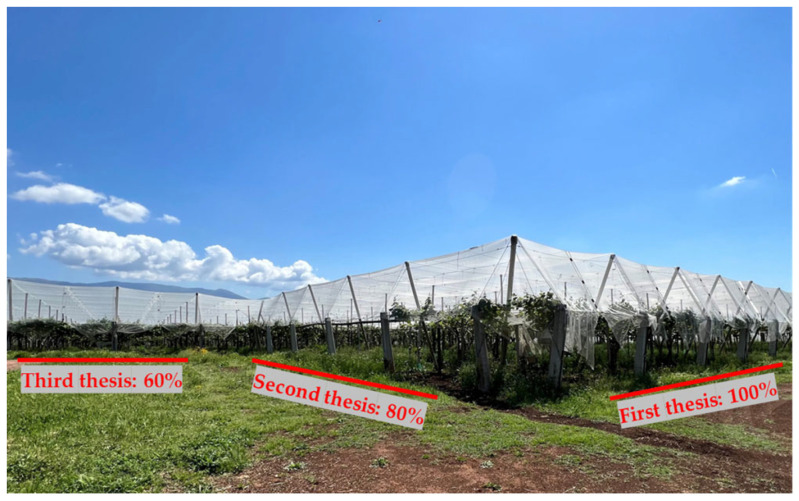
‘Soreli’ kiwifruit orchard subjected to different irrigation treatments (100%, 80%, 60%).

In the summer of 2023, an experimental plot of ‘Soreli’ kiwifruit vineyards at the “Tre Colli” farm located in Velletri (Rome, Italy) (Figure 1) was selected to study the impact of water deficit on plant health and fruit quality. This study replicated the analysis conducted in 2022 under the same conditions [13]. Each group of samples (three vines for each sample) shares the same mineral composition and cultivation practices but receives different irrigation treatments: standard drip irrigation at 50–100 L per day based on seasonal and climatic conditions (100% of full water irrigation); deficit at 80% and 60% of the full irrigation rate. The plot covers about 1 hectare and contains approximately 700 vines, with plants spaced 4.0 m between rows and 3.0 m apart within rows.

### 2.2. Kiwifruit Storage Conditions

**Figure 2:**
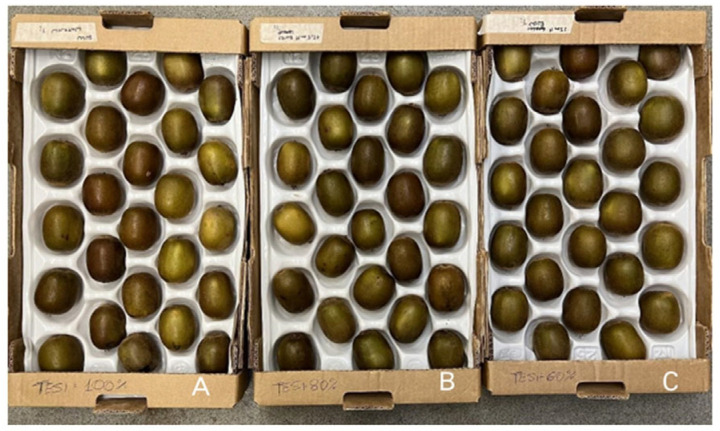
‘Soreli’ kiwifruits irrigated with three different water volumes (100% (**A**), 80% (**B**), 60% (**C**)), stored for 24 h at room temperature (20 ± 2 °C) in single-layer boxes (curing procedure).

‘Soreli’ kiwifruits were collected at 9.5 ± 1 °Brix, ensuring they were uniform in size and appearance and free from defects and disease. After harvesting, the fruits were packed in single-layer trays and swiftly transported to the Postharvest Laboratory of the Department for Innovation in Biological, Agro-Food and Forest Systems (DIBAF) at the University of Tuscia (Viterbo, Italy). Initially, the kiwifruits were kept at room temperature (20 ± 2 °C) for 24 h for the curing procedure (Figure 2).

Following this storage period, the fruits were cooled to 1 °C ± 0.5 °C with a relative humidity of 85% ± 5% in a normal atmosphere, utilizing an ethylene absorber. Analyses were conducted on fruit for seven sampling times, at the time of harvest (T0) and every 15 days of sampling time during cold storage (T1, T2, T3, T4, T5, and T6).

### 2.3. Physico-Chemical Parameters

The same twenty kiwifruits were selected and weighed 24 h after collection (T0) and during all subsequent sampling times (T1-T6) using a digital balance (Adam Equipment Co., Ltd., Milton Keynes, UK) to monitor weight loss (WL %). Additionally, 20 samples of kiwifruits for each treatment were used for destructive analyses.

**Figure 3:**
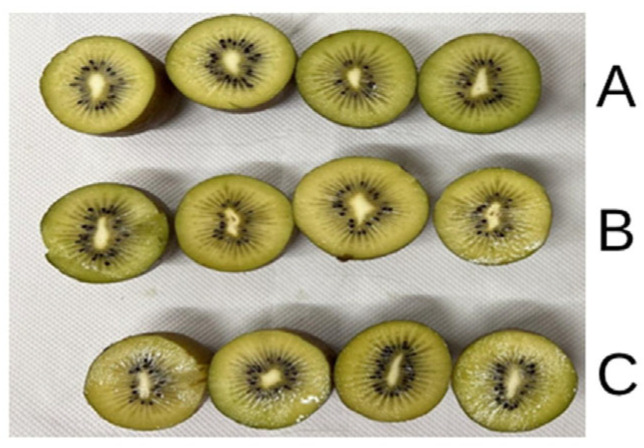
‘Soreli’ kiwifruits irrigated with three different water volumes (100% (**A**), 80% (**B**), 60% (**C**)) were cut in half for CIELab destructive colorimetric analysis.

The SSC is expressed in °Brix (%) and was evaluated using a digital refractometer (ATAGO, Palette PR-32, Tokyo, Japan). Flesh color was evaluated on half-open fruits (Figure 3) using a Minolta colorimeter (Minolta C2500; Konica Minolta, Ramsey, NY, USA) to evaluate the following parameters: L* (Lightness), a* (green to red), b* (blue to yellow), and ΔE* (the difference between two colors).

The difference (ΔE) can be classified as imperceptible (ΔE* < 1), minimal (1 ≤ ΔE* < 2), just perceptible (2 ≤ ΔE* < 5), strong difference (5 ≤ ΔE* < 12), and a different color (ΔE* ≥ 12). Fruit firmness was measured by removing a 1 mm thick slice of skin from the equatorial region of each kiwifruit and using a digital penetrometer (Model 53205; TR Turoni snc, Forlì, Italy) provided with an 8 mm tip. The firmness values were recorded in kg cm^−2^. The TA was measured by homogenizing 2 g of frozen sample tissue in distilled water and subsequently titrating each sample with an alkaline solution (NaOH, 0.1 N) until pH 8.2, following the protocol of Grasso et al. [25].

### 2.4. Bioactive Compound Content and Antioxidant Activity

Bioactive compound content was extracted using the method described by Goffi et al. [5]. POL and FLAV contents were determined using the Folin–Ciocalteu [26] and aluminum chloride colorimetric method [27], respectively, with 20 µL and 200 µL of extract. The results were expressed in mg gallic acid equivalent (GAE) 100 g^−1^ of fresh weight (FW) and mg catechin equivalent (CE) 100 g^−1^ of FW.

The ascorbic acid (AA) content in kiwifruit was measured using the method outlined by Goffi et al. [5]. The results were expressed as mg of AA 100 g^−1^ of FW.

Chlorophyll (Chl) and β-carotene (Car) contents were determined following the procedure described by Lembo et al. [13]. Pigments were extracted using acetone–hexane solution (4:6; *v*:*v*), and the absorbance of the supernatant was measured at 663 nm, 645 nm, 505 nm, and 453 nm using a spectrophotometer (Perkin Elmer Instruments Ltd., Seer Green, Beaconsfield, UK). Chl and Car results were expressed as mg 100 g^−1^ and µg 100 g^−1^ of FW, respectively.

The scavenging activity of alcoholic extracts was evaluated using the ABTS assay according to the method of Cice et al. [28]. The ABTS radical cation solution was prepared with an initial absorbance of 0.70 at 734 nm, then mixed with 5 µL of kiwifruit extracts and the absorbance was recorded after 6 min incubation. Each sample was analyzed in triplicate for technical accuracy. Trolox (TE, Merck (Darmstadt, Germany)) at concentrations of 4, 8, 16, and 32 µM was used as a positive control. Results were expressed as µL TE g^−1^ of FW.

### 2.5. Enzyme Extraction and Activity Assays

Total soluble proteins were extracted by re-suspending frozen fruit tissue powder in the extraction buffer and 5 mM ascorbic acid (used only for APX extraction) as described by Goffi et al. [5]. The homogenate was then centrifuged at 18,000× *g* for 10 min at 4 °C, and the resulting supernatant was used to assess the activity of SOD, CAT, and APX. The protein content in each crude enzyme extract was determined by the Bradford assay [29], using bovine serum albumin as a standard.

#### 2.5.1. CAT and APX Activity

CAT activity (EC 1.11.1.6) was measured using the reaction mixture described by Goffi et al. [5] for ‘Soreli’ kiwifruit. The reaction was initiated with the addition of H_2_O_2_, and the breakdown of H_2_O_2_ was tracked by monitoring the decrease in absorbance at 240 nm. Specific activity was calculated as the molar rate of H_2_O_2_ decomposition, expressed in µmol g^−1^ of FW.

APX activity (EC 1.11.1.11) was measured following the method by Goffi et al. [5]. The reaction was initiated by adding H_2_O_2_, and ascorbic acid oxidation was monitored by observing the decrease in absorbance at 290 nm. Specific activity was expressed as the molar rate of ascorbate oxidation in µmol g^−1^ of FW.

#### 2.5.2. SOD Activity

SOD activity (SOD, EC 1.15.1.1) was measured based on its inhibition of the photochemical reduction in nitro blue tetrazolium (NBT) in the presence of riboflavin, as outlined by Goffi et al. [5]. The reaction was evaluated at 560 nm and one unit of SOD activity was defined as the amount of enzyme required to inhibit NBT reduction by 50% under these conditions, with specific activity expressed as U g^−1^ of FW.

### 2.6. Hydrogen Peroxide Content Determination

Hydrogen peroxide content was measured following the method described by Alexieva et al. [30]. The reaction mixture contained 0.1% trichloroacetic acid and 1 mol L^−1^ potassium iodide and 300 μL of crude enzyme extract, and the absorbance of the mixture was recorded at 390 nm. Results were reported as nmol H_2_O_2_ kg^−1^ of FW.

### 2.7. Statistical Analysis

For physical, chemical, and enzymatic analyses, the results are given as mean values ± standard errors. Statistical comparisons between samples were performed with one-way analysis of variance (ANOVA), followed by Tukey’s test at a 5% significance. The mean values were considered significantly different when the *p* value was smaller than 0.05 (*p* < 0.05) and were marked with different letters.

A principal component analysis (PCA) was applied to describe the influence of different irrigation treatments on quality parameters and antioxidant systems to identify the components responsible for the main variations in the dataset. All analyses were performed using the SPSS software package, version 20.0 (SPSS Inc., Chicago, IL, USA).

## 3. Results and Discussion

### 3.1. Effect of Different Irrigations on Physical and Chemical Parameters of ‘Soreli’ Kiwifruit

The ‘*Soreli*’ kiwifruit plants began flowering during the last ten days of May, and harvest took place at the end of September. The season was characterized by generally high summer temperatures, reaching a maximum of 32–33 °C, while rainfall occurred regularly throughout the period. Harvest yields per plant were similar across treatments, averaging 70–80 kg, indicating that the different irrigation regimes did not affect overall fruit production.

A significant factor contributing to kiwifruit deterioration during storage is the fruit’s water loss [31]. In our experiment, no significant differences in WL values between different irrigation conditions have been found, except at T2 (Table 1).

WL increased over time and, at the end of the storage, WL reached approximately 5% for all samples. These results are confirmed by further investigations carried out for the WL of kiwifruits [13,23,32] and other studies conducted on different types of fruit, such as apricot [33] and pomegranate [34], in which comparable findings were observed regarding the influence of irrigation on WL.

The measurement of flesh firmness using a penetrometer shows a decrease during the storage period, as expected for ripening fruits [35,36,37]. Initial values (T0) were the highest, with a firmness of approximately 8 kg cm^−2^, whereas by T6, firmness declined to below 1 kg cm^−2^, indicating a highly ripe and softened fruit (no more marketable), with no significant difference between samples (Table 1). During the early sampling times (T0-T2), no significant differences were observed between the different irrigation conditions. However, at T3, T4, and T5, kiwifruits irrigated with 60% water tended to maintain greater firmness compared to other treatments, as indicated by some studies on different fruit crops; meanwhile, kiwifruits irrigated with 80% water show a similar behavior compared to the full irrigation (100%). It was observed that peach fruits from trees subjected to deficit irrigation were firmer than control fruits but did not differ in weight and diameter [33,38]. This could suggest that kiwifruits subjected to water deficit irrigation retain greater firmness during storage, possibly due to reduced water loss, enhanced structural integrity at the cellular level, and the accumulation of protective compounds. These adaptations strengthen the fruit’s cellular structure, slowing softening processes and preserving firmness over time [39].

The SSC increased progressively for all treatments during storage, which indicates a natural process of ripening and accumulation of sugars in kiwifruits, as already shown by other studies [40,41]. Overall, the maturation progression does not show significant major differences between the samples (Table 1). The works carried out by Alcobendas et al. [38] and Navarro et al. [42] on mandarin fruit confirmed our results: water deficit stress tends to accumulate higher levels of carbohydrates than those without water stress.

TA values were similar in the three irrigation treatments, as shown by Conesa et al. [36] in nectarine fruit, and they did not exhibit significant changes during cold storage. Also, Goffi et al. [5] observed no significant changes in TA content of ‘Soreli’ kiwifruits during cold storage.

Overall, all measured quality parameters followed expected ripening trends, with minor differences between irrigation treatments. These results suggest that moderate water deficits do not negatively affect the postharvest quality of ‘Soreli’ kiwifruits.

**Figure 4:**
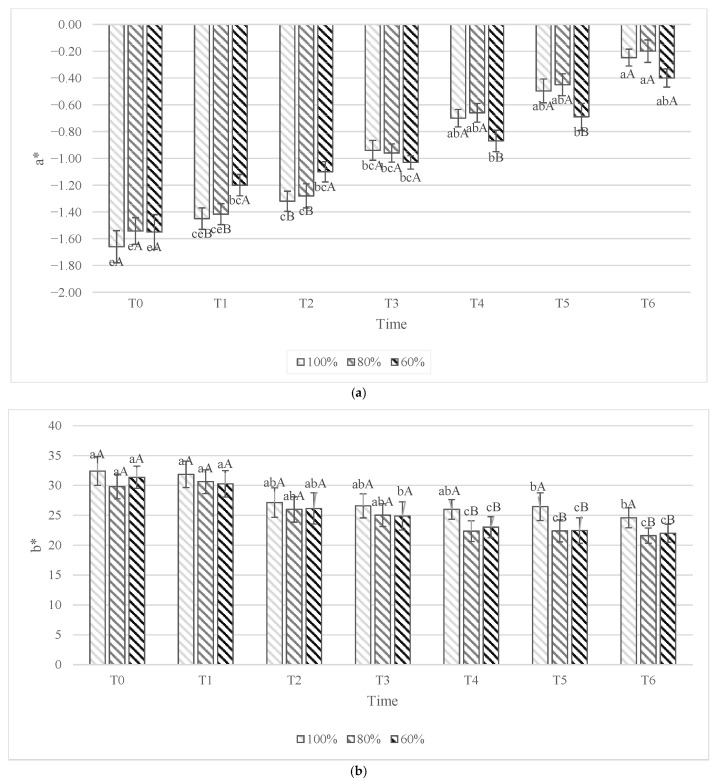
Color parameters a*, Green–Red axis (**a**); b*, Blue–Yellow axis (**b**) in ‘Soreli’ kiwifruits irrigated with three different water volumes (100%, 80%, 60%) at 0 days (T0), 15 days (T1), 30 days (T2), 45 days (T3), 60 days (T4), 75 days (T5), and 90 days (T6) of cold storage at 1 °C. Capital letters indicate comparisons between different treatments of fruits at each specific time. Lowercase letters reflect comparisons between different storage times for each sample fruit of the same treatment. According to the Tukey test (*p* < 0.05), significant differences between means are indicated with different letters.

The flesh color of yellow kiwifruits showed a decrease during 90 days of storage for the CIELab coordinates a*, b* (Figure 4a,b), and L*. The same trend was observed in ‘Soreli’ kiwifruits in a previous study by Grasso et al. [25].

The a* parameter in kiwifruits tends to lower negative values during storage, regardless of different irrigation volumes (Figure 4a). This trend suggests a reduction in green intensity, potentially indicating a gradual loss of chlorophyll as the fruit ripens, which also happens in the leaves of plants [43]. During the storage, as observed for the chlorophyll content behavior (Table 2), the color parameter shows higher values of green for the 80% and 100% samples in T1 and T2, while subsequently, the kiwifruit subjected to 60% irrigation treatments exhibited a* values more negative compared to those irrigated with 80% and 100% water up to T5.

Overall, the yellow color (b* parameter) shows a trend to decrease in all samples regardless of the different treatments. In the samples irrigated with 100% of water, the b* values from T4 to T6 are significantly higher than in the other samples. This observation seems to confirm that adequate water availability promotes the development of desirable yellow coloration in the fruit across the final storage times (Figure 4b).

These observations suggest that the water deficit seems to induce possible changes in flesh color. In this sense, in peach fruit it was observed that higher irrigation levels correlate with a shift toward a more favorable coloration profile, characterized by enhanced yellow hues [38].

ΔE* facilitates accurate assessment and enhances our understanding of the color changes in fruit that occur during cold storage time [44]. No differences were noted within the three samples: all kiwifruits showed a different color (ΔE* ≥ 12) compared to the initial storage time.

In general, flesh color changes followed typical ripening patterns, with minor variations between irrigation treatments. These results suggest that moderate water deficits may slightly affect color development, but do not prevent the overall maturation and yellowing of ‘Soreli’ kiwifruits during storage.

### 3.2. Effect of Different Irrigations on Bioactive Compounds of ‘Soreli’ Kiwifruit

The literature indicates that kiwifruit AA content may reach its maximum value during the full fruit ripening phase and subsequently stabilize or decrease during storage time [25,45,46]. This aligns with our results shown in Table 2, in which, regardless of the three samples, an increase in vitamin C content occurred during the initial periods of storage, followed by subsequent stabilization or slight decrease. The fruits from plants irrigated with 100%, 80%, and 60% of water reach their maximum AA content at T3, T4, and T2, respectively. The fluctuation of antioxidants’ behavior, specifically AA content, can be affected by a number of different conditions, such as temperature, availability of sunlight, agronomic practices, and irrigation techniques [25].

In the study conducted by Navarro et al. [42], the AA content was higher in mandarin fruits harvested from plants subjected to water stress compared to control fruits. Additionally, in tomatoes, it was observed that reducing water irrigation increased the fruit AA content compared to the fully irrigated control [47].

In addition to the high content of AA, kiwifruits are also rich in other antioxidants, such as phenolic compounds, flavonoids, and carotenoids. These latter compounds are responsible for the yellow color of flesh and offer various health benefits. They can help scavenge ROS, slow down the aging process, prevent cardiovascular and eye diseases, and strengthen the immune system in humans [48].

As previously observed in the study by Grasso et al. [25] on ‘Soreli’ kiwifruits, our experiment shows a clear decreasing trend in carotenoid content in the fruit flesh across all treatments during the storage period (Table 2).

Fruits harvested from plants with 100% irrigation initially (T0 and T1) show the highest levels of β-carotene, suggesting that good water availability may promote early carotenoid synthesis (Table 2). Fruits irrigated at 80% and 60% exhibit a smaller decline in carotenoids, indicating that moderate watering may help maintain carotenoid levels, though it may also limit synthesis. Otherwise, studies on cherry and goji berry varieties have shown that reduced irrigation can increase total CAR content, while results in tomato varieties under water deficit stress are consistent with our findings on ‘Soreli’ kiwifruit [49,50].

Chl is found in various plant organs, including unripe fruits, and typically undergoes degradation during the ripening process [51]. As can be seen in Table 2, the Chl content in the ‘Soreli’ yellow fruit’s flesh tends to decrease during the storage times. Photosynthetic pigment values appear to be higher in fruits from the plants irrigated at 80% and 100% during the early stages of storage (T1 and T2). Fruits from plants irrigated at 60% show an initial lower Chl level compared to the other samples, but Chl concentration remains consistent at T4, T5, and T6, suggesting a possible physiological response to water stress. To confirm that “regulated” water stress can also exhibit a positive effect on Chl content, Rana Yashwant Singh Parmar [52] evaluated the chlorophyll stability index (CSI) of kiwifruit cultivars irrigated with different volumes of water and showed that the water-deficient cultivars underwent a significant increase in CSI compared to the control, suggesting greater stability to Chl degradation under stress conditions. Furthermore, the later decline may result from natural Chl degradation and shifts in metabolic processes [5,8]. A reduction in Chl content during storage has also been verified by other kiwifruit CVs as well as different fruits [25,51,53,54,55,56].

**Figure 5:**
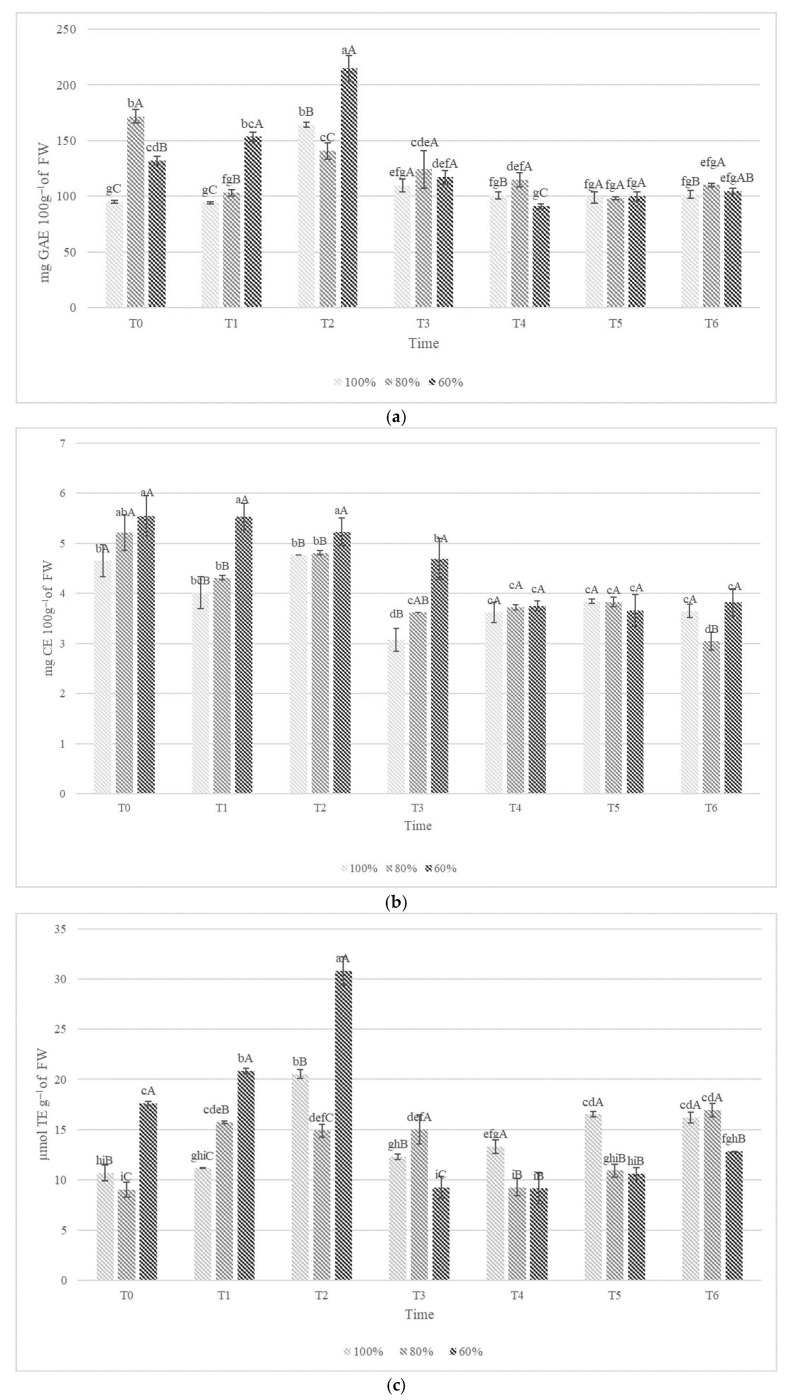
Polyphenol content (POL) (**a**); Flavonoid content (FLAV) (**b**); ABTS assay (**c**) in ‘Soreli’ kiwifruits irrigated with three different water volumes (100%, 80%, 60%) at 0 days (T0), 15 days (T1), 30 days (T2), 45 days (T3), 60 days (T4), 75 days (T5), and 90 days (T6) of cold storage at 1 °C. Capital letters indicate comparisons between different treatments of fruits at each specific time. Lowercase letters reflect comparisons between different storage times for each sample fruit of the same treatment. According to the Tukey test (*p* < 0.05), significant differences between means are indicated with different letters.

The POL and FLAV contents and ABTS assay in kiwifruit, measured at various times (T0 to T6) under different irrigation levels, are shown in Figure 5.

Kiwifruit is a rich source of POL in green- and yellow-fleshed kiwifruit varieties, ranging in concentrations from 58.45 to 152 mg GAE 100 g^−1^ of FW [57]. In our study, the POL content showed significant differences at harvest, with the highest levels observed in fruit harvested from plants irrigated at 80% water irrigation (Figure 5a). In the early stages, POL levels generally increase under reduced irrigation, with the highest values consistently observed in the 60% irrigation group. Notably, at both T1 and T2, the polyphenol content in the 60% irrigation group peaks significantly, exceeding 200 mg GAE 100 g^−1^ FW in the T2 sample, indicating that a moderate water deficit positively influences polyphenol accumulation. Starting from T3, the results show smaller differences between the three different samples.

The FLAV levels also exhibit an upward trend with reduced irrigation. The 60% irrigation treatment again leads to the highest FLAV levels, particularly at T1, T2, and T3 with the respective values of 5.53, 5.23, and 4.69 mg CE 100 g^−1^ of FW (Figure 5b). This suggests that water stress may enhance FLAV synthesis in the fruit.

In general, samples irrigated at 80% and 100% show a similar pattern for both POL and FLAV content, with a decreasing trend over time. Our results are consistent with the findings of Jin et al. [58], who demonstrated that four water deficit treatments improve POL and FLAV content in tomato fruit.

For the ABTS assay, the 60% irrigation treatment showed the highest antioxidant activity, with a marked peak at T0, T1, and T2. This peak aligned with the general trends observed for POL and FLAV content, suggesting a positive correlation between water stress and antioxidant capacity (Figure 5c).

Ebel and coworkers have demonstrated that the irrigation regime increased the bioactive compound content and antioxidant activity in different apple cultivars [59].

Overall, the results indicate that the lowest irrigation level (60%) enhances the accumulation of bioactive compounds and promotes higher antioxidant activity in ‘Soreli’ kiwifruits during storage. These findings suggest that regulated water deficit stress may positively influence fruit quality and nutritional value without compromising overall ripening and storage performance.

### 3.3. Effect of Different Irrigations on the Antioxidant Enzymatic System of ‘Soreli’ Kiwifruit

The activity of key antioxidant enzymes, SOD, CAT, and APX, and the H_2_O_2_ content in ‘Soreli’ kiwifruit under three irrigation levels (100%, 80%, and 60%) at different storage times (T0 to T6) are reported in Table 3.

SOD activity is highest in fruits under the 60% irrigation treatment, especially at T2, reaching the highest value (3.03 ± 0.07 U g^−1^ of FW). In the early stages, SOD activity significantly increases also for 100% and 80% irrigation treatment, then it decreases gradually over storage time for all treatments.

Similarly to SOD, CAT activity is highest in kiwifruit under the 60% irrigation regime. However, all treatments show a significant decrease during cold storage.

APX activity is also highest under the 60% irrigation treatment across all time points. Additionally, for this activity, the results show a significant reduction for all samples over time.

Several studies on the reduction in water supply and antioxidant enzyme activity in fruits have shown that water stress can significantly influence the enzymatic antioxidant response, activating defense mechanisms to combat oxidative stress. Enzymes such as SOD, CAT, and APX play essential roles in reducing oxidative damage by neutralizing ROS, including H_2_O_2_, which increases under drought stress [60,61]. These enzymes are crucial in maintaining fruit quality, as oxidative damage can degrade cellular structures and reduce nutritional value [62]. In tomatoes, water deficit has been shown to enhance the activity of SOD and CAT, which helps to protect the fruit’s cellular integrity and maintain its nutritional quality even under challenging environmental conditions [63,64].

Furthermore, in pomegranate fruit, deficit irrigation increased antioxidant activity, potentially due to enhanced enzymatic antioxidants like SOD, CAT, and APX, protecting against oxidative damage [65]. In grapes, regulated deficit irrigation was linked to increased SOD and APX activities, helping vines manage oxidative stress and improving fruit resilience under water stress [66] as well as water-limited conditions led to higher antioxidant enzyme activities in olives, with enzymes like CAT and APX being particularly responsive to deficit irrigation [67].

Our results indicate that the lowest irrigation level (60%) enhances the activity of key antioxidant enzymes in ‘Soreli’ kiwifruits. These findings suggest that regulated water deficit stress can activate defense mechanisms against oxidative damage, helping to maintain fruit quality and nutritional value during postharvest storage.

### 3.4. PCA of Physico-Chemical and Antioxidant System in ‘Soreli’ Kiwifruits Irrigated with Three Different Water Volumes

PCA is a statistical technique that can simplify the complexity of multivariate data by reducing its dimensions, making it easier to observe trends and relationships. In this study, PCA was applied to analyze the effects of different irrigation levels on various measured characteristics in kiwifruit. By condensing the data into principal components, PCA highlights which variables are most influenced by irrigation treatments. This approach facilitates the visualization of how different irrigation levels correlate with changes in key physiological and biochemical traits of the fruit.

**Figure 6:**
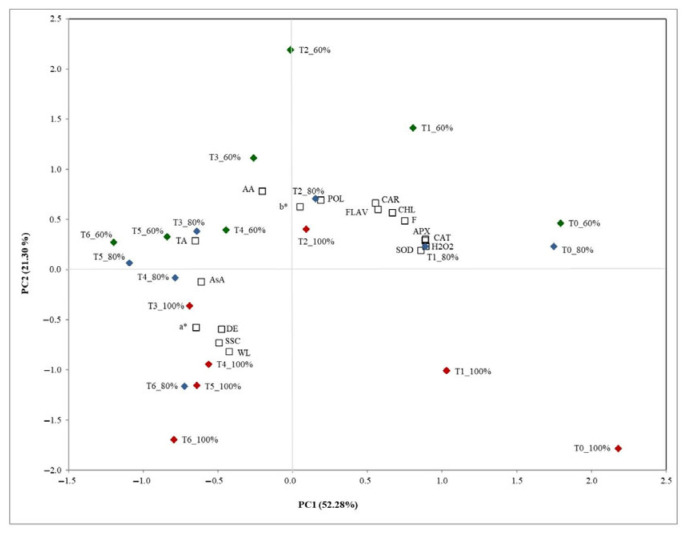
Two-dimensional principal component analysis (PCA) plot of physico-chemical and antioxidant system in ‘Soreli’ kiwifruits irrigated with three different water volumes (100%, 80%, 60%) at 0 days (T0), 15 days (T1), 30 days (T2), 45 days (T3), 60 days (T4), 75 days (T5), and 90 days (T6) of cold storage at 1 °C (superoxide dismutase (SOD), catalase (CAT), ascorbate peroxidase (APX) and H_2_O_2_ content (H_2_O_2_), chorophylls (CHL), carotenoids (CAR), ascorbic acid (AsA), polyphenol content (POL), flavonoid content (FLAV), antioxidant activity (AA), firmness (F), solid soluble content (SSC), titratable acidity (TA), color change (DE), colorimetric coordinates (a* and b*), weight loss (WL)).

PCA plot (Figure 6) illustrated the distribution of kiwifruit samples subjected to different irrigation levels (100%, 80%, and 60%) over the storage period (T0 to T6), based on principal components PC1 (52.28% of variance) and PC2 (21.30% of variance).

Samples from higher irrigation levels (100%) generally cluster toward the lower quadrants, especially in earlier time points (T0, T1) toward the lower right, suggesting that these samples are distinct in their characteristics from those under reduced irrigation. Over time, 100% irrigation samples tend to shift along PC1, particularly between T1 and T6, likely reflecting a progressive reduction in traits like antioxidant enzyme activities. In the early stages of cold storage, the 80% and 60% irrigation treatments cluster in the upper right quadrant, characterized by the main bioactive compounds and antioxidant enzyme activities.

In the later stages, the 100% and 80% irrigation treatments cluster in the lower left quadrant. In comparison, the 60% irrigation treatment is found in the upper left quadrant, characterized by higher antioxidant activity and TA.

The middle irrigation level (80%) samples were more central with progressive shifts toward the left, indicating a moderate response in biochemical attributes compared to the other treatments.

Overall, the PCA indicates that different irrigation levels induce distinct profiles in kiwifruit, with water deficit (60%) treatments enhancing antioxidant-related parameters, while fully irrigated samples (100%) show a consistent shift towards lower values of these parameters over time. This shift underscores the potential of moderate water stress to promote antioxidant defenses in stored kiwifruit.

Several studies have applied PCA to analyze crop characteristics under different irrigation conditions, focusing on optimizing water usage and improving yield efficiency, demonstrating how PCA can be effectively applied to optimize irrigation practices, providing valuable insights into water usage efficiency and crop performance under varying irrigation strategies [68,69].

## 4. Conclusions

In conclusion, the reduction in irrigation water for the ‘Soreli’ kiwifruit cultivar presents considerable commercial, environmental, and qualitative advantages.

Our findings indicate that a controlled reduction in preharvest water enhances antioxidant enzyme levels, specifically APX, SOD, and CAT, in postharvest fruits. This suggests that reduced irrigation may activate defense mechanisms against oxidative stress.

Moreover, fruits irrigated at 60% of full water supply demonstrated increased resistance to firmness loss, higher accumulation of soluble solids, and greater color stability. Consistent with these observations, the levels of polyphenols, flavonoids, and total antioxidant activity were higher in fruits with 60% irrigation.

This evidence suggests that an appropriate water deficit allows the preservation of quality attributes for longer periods, positively impacting antioxidant retention and maintaining the fruit’s quality and nutritional value during storage. These results underscore how effective water management can serve as a viable strategy to enhance postharvest fruit quality, promoting the retention of bioactive compounds, color, and firmness while boosting the antioxidant capacity of ‘Soreli’ CV. Therefore, moderate irrigation emerges as a sustainable and beneficial approach to improve the quality and storability of ‘Soreli’ kiwifruits. Although this study was conducted over two seasons in a single location, providing useful insights into the effects of regulated deficit irrigation on ‘*Soreli*’ kiwifruit quality, further research is needed to confirm these findings. Future studies could explore the effects of further reducing the irrigation regime to better understand its impact on fruit quality, antioxidant activity, and stress responses.

## Figures and Tables

**Table 1:** Physico-chemical parameters (Weight Loss, WL; Firmness; Solid Soluble Content, SSC) in ‘Soreli’ kiwifruits irrigated with three different water volumes (100%, 80%, 60%) at 0 days (T0), 15 days (T1), 30 days (T2), 45 days (T3), 60 days (T4), 75 days (T5), and 90 days (T6) of cold storage at 1 °C.

Parameter	Treatment	T0	T1	T2	T3	T4	T5	T6
**WL** **(%)**	100%	-	0.63 ± 0.17 (dA)	1.47 ± 0.26 (cB)	4.15 ± 0.24 (bA)	4.30 ± 0.29 (abA)	4.45 ± 0.33 (abA)	4.54 ± 0.43 (abA)
	80%	-	0.78 ± 0.18 (dA)	2.37 ± 0.32 (cA)	4.65 ± 0.85 (abA)	4.75 ± 0.93 (abA)	4.90 ± 0.85 (abA)	5.15 ± 0.87 (aA)
	60%	-	0.69 ± 0.21 (dA)	1.83 ± 0.15 (cA)	4.10 ± 0.67 (bA)	5.03 ± 0.35 (aA)	5.11 ± 0.33 (aA)	5.26 ± 0.29 (aA)
**Firmness** **(kg cm^−2^)**	100%	8.10 ± 0.42 (aA)	6.23 ± 0.52 (bA)	5.74 ± 0.38 (bA)	4.70 ± 0.48 (cB)	3.01 ± 0.35 (dB)	1.48 ± 0.26 (eC)	0.74 ± 0.12 (fA)
	80%	8.04 ± 0.44 (aA)	6.37 ± 0.45 (bA)	5.39 ± 0.50 (bcA)	5.15 ± 0.55 (cAB)	2.99 ± 0.39 (dB)	1.97 ± 0.16 (eB)	0.96 ± 0.17 (fA)
	60%	8.02 ± 0.16 (aA)	6.69 ± 0.49 (bA)	6.21 ± 0.56 (bA)	6.03 ± 0.60 (bA)	5.49 ± 0.45 (bA)	2.89 ± 0.49 (dA)	0.82 ± 0.22 (fA)
**SSC** **(°Brix)**	100%	10.22 ± 0.57 (fA)	12.36 ± 0.34 (dA)	13.85 ± 0.39 (cA)	13.84 ± 0.42 (cA)	14.29 ± 0.46 (bA)	14.32 ± 0.40 (bB)	14.72 ± 0.45 (bAB)
	80%	9.98 ± 0.62 (fA)	12.38 ± 0.36 (dA)	12.97 ± 0.34 (cdAB)	14.29 ± 0.37 (bcA)	14.55 ± 0.35 (bA)	14.57 ± 0.38 (bAB)	14.56 ± 0.59 (bB)
	60%	9.82 ± 0.41 (fA)	11.67 ± 0.31 (eB)	12.58 ± 0.44 (dB)	13.75 ± 0.38 (cA)	14.48 ± 0.35 (bA)	15.34 ± 0.39 (aA)	15.62 ± 0.30 (aA)

Note: Capital letters indicate comparisons between different treatments of fruits at each specific time. Lowercase letters reflect comparisons between different storage times for each sample fruit of the same treatment. According to the Tukey test (*p* < 0.05), significant differences between means are indicated with different letters within the same column or row.

**Table 2:** Ascorbic acid (AA), β-carotene (Car), and chlorophyll (Chl) contents in ‘Soreli’ kiwifruits irrigated with three different water volumes (100%, 80%, 60%) at 0 days (T0), 15 days (T1), 30 days (T2), 45 days (T3), 60 days (T4), 75 days (T5), and 90 days (T6) of cold storage at 1 °C.

Parameter	Treatment	T0	T1	T2	T3	T4	T5	T6
**AA** **(mg 100 g^−1^ of FW)**	100%	38.84 ± 0.86 (efA)	48.61 ± 0.72 (cdA)	55.46 ± 0.91 (bA)	62.26 ± 1.18 (aA)	58.02 ± 0.85 (bA)	55.56 ± 1.70 (bA)	56.11 ± 0.80 (bA)
	80%	36.82 ± 0.83 (fB)	45.4 ± 0.81 (dB)	50.18 ± 0.76 (cB)	54.14 ± 1.76 (bB)	57.41 ± 1.13 (bA)	55.64 ± 1.90 (bA)	50.89 ± 0.93 (cB)
	60%	40.49 ± 1.30 (eA)	46.62 ± 1.02 (dB)	54.77 ± 1.64 (bA)	51.25 ± 2.23 (bcB)	47.51 ± 1.39 (dB)	52.9 ± 1.97 (bcA)	50.38 ± 1.50 (cB)
**Car** **(mg 100 g^−1^ of FW)**	100%	29,8 ± 0.98 (aA)	22.42± 0.75 (bA)	11.23 ± 0.59 (dA)	5.43 ± 0.53 (fB)	3.22 ± 0.38 (gA	1.52 ± 0.35 (iB)	1.10 ± 0.25 (iA)
	80%	23.10 ± 1.22 (bB)	14.63 ± 0.66 (cB)	10.89 ± 0.65 (dA)	6.89 ± 0.58 (eA)	3.69 ± 0.43 (gA)	2.18 ± 0.31 (hA)	1.32 ± 0.33 (iA)
	60%	22.60 ± 0.88 (bB)	15.02 ± 0.77 (cB)	10.55 ± 0.62 (dA)	6.59 ± 0.49 (eA)	3.60 ± 0.35 (gA)	2.33 ± 0.36 (hA)	1.26 ± 0.28 (iA)
**Chl** **(µg 100 g^−1^ of FW)**	100%	255.04 ± 7.53 (aA)	245.22 ± 7.69 (bA)	220.42 ± 8.45 (abA)	176.87 ± 7.62 (bA)	138.78 ± 6.13 (cB)	132.45 ± 7.09 (cA)	103.63 ± 6.89 (dA)
	80%	268.72 ± 11.50 (aA)	254.36 ± 10.73 (aA)	221.08 ± 7.23 (abA)	188.06 ± 8.85 (bA)	140.89 ± 8.44 (cB)	128.56 ± 7.79 (cB)	75.44 ± 8.53 (eB)
	60%	254.24 ± 9.83 (aA)	221.52 ± 8.53 (abB)	196.99 ± 8.85 (bB)	179.52 ± 7.53 (bA)	163.22 ± 7.86 (bcA)	147.66 ± 8.63 (cA)	108.86 ± 6.62 (dA)

Note: Capital letters indicate comparisons between different treatments of fruits at each specific time. Lowercase letters reflect comparisons between different storage times for each sample fruit of the same treatment. According to the Tukey test (*p* < 0.05), significant differences between means are indicated with different letters within the same column or row.

**Table 3:** Superoxide dismutase (SOD), catalase (CAT), ascorbate peroxidase (APX) activity, and H_2_O_2_ content in ‘Soreli’ kiwifruits irrigated with three different water volumes (100%, 80%, 60%) at 0 days (T0), 15 days (T1), 30 days (T2), 45 days (T3), 60 days (T4), 75 days (T5), and 90 days (T6) of cold storage at 1 °C.

Parameter	Treatment	T0	T1	T2	T3	T4	T5	T6
SOD (U g^−1^ of FW)	100%	1.64 ± 0.08 (klC)	1.92 ± 0.06 (hijB)	2.12 ± 0.09 (efghC)	1.88 ± 0.06 (hijkC)	1.67 ± 0.0 (jklC)	1.51 ± 0.03 (lmC)	1.31 ± 0.04 (mC)
	80%	1.98 ± 0.06 (ghiB)	2.63 ± 0.17 (bcA)	2.75 ± 0.10 (bcB)	2.28 ± 0.04 (efB)	2.09 ± 0.06 (efghB)	2.02 ± 0.05 (fghiB)	1.80 ± 0.05 (ijkB)
	60%	2.2 ± 0.05 (efgA)	2.85 ± 0.11 (abA)	3.03 ± 0.07 (aA)	2.86 ± 0.09 (abA)	2.55 ± 0.11 (cdA)	2.31 ± 0.10 (deA)	2.27 ± 0.04 (efA)
CAT (µmol g^−1^ of FW)	100%	5.07 ± 0.06 (cdefC)	4.85 ± 0.08 (fgC)	4.67 ± 0.05 (ghB)	4.3 ± 0.04 (ijB)	4.12 ± 0.04 (jkB)	3.57 ± 0.16 (lC)	3.03 ± 0.05 (mC)
	80%	5.84 ± 0.07 (abB)	5.17 ± 0.05 (cdB)	4.82 ± 0.09 (efgB)	4.24 ± 0.03 (ijB)	4.10 ± 0.02 (lkB)	4.04 ± 0.04 (jkB)	3.87 ± 0.07 (kB)
	60%	6.07 ± 0.05 (aA)	5.80 ± 0.08 (abA)	5.64 ± 0.10 (bA)	5.31 ± 0.20 (cA)	5.11 ± 0.03 (cdeA)	4.92 ± 0.04 (defgA)	4.51 ± 0.17 (hiA)
APX (µmol g^−1^ of FW)	100%	0.36 ± 0.03 (efC)	0.32 ± 0.03 (fghC)	0.29 ± 0.02 (fghiB)	0.24 ± 0.02 (ijC)	0.20 ± 0.02 (jkC)	0.17 ± 0.01 (kC)	0.13 ± 0.01 (kC)
	80%	0.47 ± 0.03 (bcB)	0.39 ± 0.02 (deB)	0.34 ± 0.02 (efgB)	0.32 ± 0.01 (fghB)	0.27 ± 0.02 (ghijB)	0.25 ± 0.03 (hijB)	0.20 ± 0.01 (jkB)
	60%	0.58 ± 0.03 (aA)	0.53 ± 0.02 (abA)	0.45 ± 0.03 (cdA)	0.40 ± 0.02 (cdeA)	0.35 ± 0.03 (efA)	0.32 ± 0.03 (fghA)	0.30 ± 0.01 (fghiA)
H_2_O_2_ (nmol kg^−1^ of FW)	100%	45.80 ± 1.59 (eC)	40.77 ± 0.45 (fghC)	37.97 ± 0.59 (jkC)	33.97 ± 0.32 (lmC)	33.80 ± 0.30 (lmC)	32.37 ± 0.45 (mC)	32.03 ± 0.40 (mC)
	80%	51.10 ± 0.26 (bB)	48.27 ± 1.07 (cdB)	45.77 ± 0.55 (eB)	42.93 ± 0.67 (fgB)	40.97 ± 0.21 (ghiB)	38.67 ± 0.25 (ijB)	35.83 ± 0.25 (klB)
	60%	55.23 ± 0.95 (aA)	51.90 ± 0.36 (bA)	49.90 ± 0.61 (bcA)	47.27 ± 0.86 (deA)	45.83 ± 0.85 (eA)	43.07 ± 0.74 (fA)	39.10 ± 0.56 (hijA)

Note: Capital letters indicate comparisons between different treatments of fruits at each specific time. Lowercase letters reflect comparisons between different storage times for each sample fruit of the same treatment. According to the Tukey test (*p* < 0.05), significant differences between means are indicated with different letters within the same column or row.

## Data Availability

The original contributions presented in this study are included in the article. Further inquiries can be directed to the corresponding author.
